# Cross-platform public judgment in a cultural heritage crisis: A topic modeling study of the Nanjing Museum donated-artifact controversy

**DOI:** 10.1371/journal.pone.0360658

**Published:** 2026-10-09

**Authors:** Wei Li, Ming Ma, Jinyue Lu

**Affiliations:** Faculty of Humanities and Social Sciences, City University of Macau, Macau, China; Universiti Malaya, MALAYSIA

## Abstract

Crises involving cultural institutions increasingly unfold across multiple platforms, where the same controversy is interpreted through different content formats, interaction structures, and community norms. This study develops distributed accountability as a function-based framework for examining how public demands for sanction, explanation, and relational repair are organized across platform environments. The study analyzes 64,349 publicly visible top-level comments from Douyin, Bilibili, and Rednote, organized into three event-anchored source-content groups concerning the Nanjing Museum donated-artifact controversy. BERTopic identified 49 substantive topics covering 42,126 comments. A higher-order thematic framework was subsequently applied and validated by two researchers, producing six substantive groupings mapped onto punitive, knowledge-based, and ethical accountability functions. Under the primary coding specification, ethical accountability accounted for the largest share of classified comments, although this absolute ranking was sensitive to the treatment of the largest disputed topic. Across event-anchored groups, donation ethics was relatively more prominent in discussion attached to the initial response. Investigation-oriented and personalized-blame themes were relatively more prominent in later groups. Cross-platform differences were more stable. Douyin showed a relatively stronger punitive profile, and Bilibili showed a relatively stronger knowledge-based profile across alternative coding specifications. The overall platform–function association remained small. These findings indicate that platform differences concern the relative organization of accountability discourse, not the presence or absence of particular accountability functions. The study extends research on platformized public judgment and institutional crisis governance by showing how functionally differentiated accountability demands coexist within a shared institutional crisis.

## Introduction

Contemporary cultural institutions operate under sustained public scrutiny. When a controversy emerges, institutional statements, journalistic reports, user comments, and secondary interpretations circulate together within continuous and asynchronous platform environments [1,2]. Affective expression can gain visibility before factual accounts stabilize, and negative emotions can circulate rapidly across communicative boundaries [3]. Comment-based interaction allows temporary interpretive publics to form around shared evaluations of institutional conduct [4]. The consequences are pronounced for museums, whose authority rests on public confidence in custodial responsibility, procedural integrity, and public mission [5,6]. Scrutiny of a single incident can extend into judgments about professional conduct, decision-making, and governance capacity [7,8]. A platform-mediated controversy involving a cultural institution can develop into a wider challenge to institutional legitimacy.

Existing scholarship explains separate parts of this process. Research on platform crisis discourse has examined information diffusion, issue visibility, and affective reaction to controversy [9–11]. Crisis communication research has analyzed responsibility attribution, reputational threat, and organizational response strategy [12–14]. Institutional theory and museum studies have examined how organizational legitimacy is maintained and repaired [15,16]. These literatures provide few analytical tools for distinguishing among the different demands for institutional responsiveness that coexist across platform environments during a single crisis. They treat public reaction largely as a single quantity of reputational pressure and give limited attention to its functional composition.

Distributed accountability is developed here to describe the public-side organization of accountability pressure under these conditions. The concept refers to the concurrent production of functionally differentiated demands for institutional responsiveness across platform environments. Punitive accountability concerns culpability, investigation, sanction, and visible consequence. Knowledge-based accountability concerns explanation, evidence, procedural reasoning, documentation, and expertise. Ethical accountability concerns moral obligation, restitution, apology, relational repair, and trust. These functions remain analytically independent of any particular platform, and their relative salience varies across platform-specific corpora.

The concept is distinct from networked accountability. Networked accountability maps relations among actors, forums, and oversight arrangements, and answers the question of who holds whom to account. Distributed accountability identifies what public accountability demands ask for and how those demands are distributed across communicative environments. The distinction makes two things analyzable that a relational mapping leaves aggregated. One is the coexistence of incommensurable demands within a single controversy, where sanction, evidentiary explanation, and relational repair cannot be satisfied by one institutional response. The other is the relative salience of each demand as an empirical property of a communicative environment, not a fixed attribute of a platform.

The controversy centered on the whereabouts and handling of paintings from the Pang family’s 1959 donation to the Nanjing Museum. Public attention intensified after the Jiangnan Chun scroll attributed to Qiu Ying, one of the donated works, appeared in a Beijing auction preview in 2025, prompting questions about how donated paintings had left or become unaccounted for in the museum’s collection. The dispute also raised questions about earlier authenticity assessments and the procedures through which several donated works had been removed from the collection and transferred into market circulation. Media reporting intensified public attention in December 2025, and the museum issued a statement on 17 December. On 23 December 2025, the National Cultural Heritage Administration announced that a working group had arrived in Nanjing [17]. On the same day, provincial authorities established an investigation group involving discipline inspection, public security, cultural heritage, and other relevant agencies [18]. Subsequent developments kept the conduct of the investigation under sustained public scrutiny. The official findings, released on 9 February 2026, attributed irregularities in the 1990s disposal process to former museum leadership and announced that twenty-four individuals would be investigated and dealt with in accordance with applicable rules and law [19]. The museum issued a public apology on the same day.

The controversy connected questions about the location and authenticity of donated works with concerns about collection management, donor relations, procedural transparency, and public trust. Discussion developed concurrently on Douyin, Bilibili, and Rednote, which differ in content form, interaction structure, and established modes of public expression. The case therefore allows examination of how factual scrutiny, responsibility claims, and legitimacy evaluation are organized across several platform-specific corpora within one institutional crisis.

The analysis uses 64,349 publicly visible top-level comments organized into three event-anchored source-content groups. Content group A contains comments attached to source content associated with the museum’s initial statement and the regulatory intervention of 17 to 23 December 2025. Content group B contains comments attached to source content associated with the continuing investigation and related developments between 27 and 30 December 2025. Content group C contains comments attached to source content associated with the official findings, the apology, and subsequent discussion between 9 and 27 February 2026. These groups are not comment-posting time bins. Comment posting dates overlap across groups, and comments attached to content from earlier phases continue to appear after later anchor events. The comparison identifies differences among event-anchored discourse settings and does not estimate a temporal progression of public opinion.

The first analytical task concerns the substantive organization of public judgment. Topic modeling allows recurrent concerns to emerge from the corpus without reducing comments to a single sentiment measure and without imposing the three accountability functions directly on individual comments. The analysis separates model-derived topic discovery from researcher-developed higher-order coding. First-order topics were identified inductively through topic modeling. The higher-order thematic framework was developed through an earlier stage of independent coding by two coders followed by consensus discussion, and was subsequently fixed for a separate independent validation round. The three-layer framework provides an interpretive lens for considering how the resulting thematic groupings relate to affective expression, responsibility attribution, and legitimacy questioning; these layers are not treated as separately measured dimensions.

**RQ1. What first-order topics are identified in cross-platform comments on a cultural institution’s crisis, and how are these topics organized within the higher-order thematic framework?**

Source content associated with different event phases may attract distinct combinations of factual scrutiny, responsibility attribution, and legitimacy concern. Comparing event-anchored content groups shows how the composition of public judgment differs across discussions attached to an initial institutional response, an ongoing investigation, and formal findings. The theoretical question is how the composition of thematic concerns differs across event-anchored discourse settings associated with different phases of the same institutional crisis.

**RQ2. How does the relative salience of thematic groupings vary across event-anchored content groups?**

Platform research indicates that content format, interface structure, distribution system, and community convention shape the conditions under which public expression becomes visible [20,21]. Cross-platform comparison can assess whether punitive, knowledge-based, and ethical accountability occupy different relative positions across platform-specific corpora. The theoretical question is whether accountability functions vary in patterned ways across communicative environments while remaining analytically independent of any particular platform.

**RQ3. How does the relative salience of punitive, knowledge-based, and ethical accountability vary across the Douyin, Bilibili, and Rednote corpora?**

The study makes three contributions. Theoretically, it distinguishes functionally differentiated public accountability demands from the formal actor-forum relationships that accountability scholarship has mainly addressed, and it specifies what that distinction makes visible in a platformized crisis. Empirically, it examines how thematically distinct forms of public judgment coexist within one controversy and how punitive, knowledge-based, and ethical accountability are organized across three platform-specific corpora. Practically, it supports institutional responses calibrated to distinct forms of accountability pressure without assuming that each platform corresponds to a single accountability function.

## Literature review

This review examines how institutional legitimacy, accountability, responsibility attribution, and platformization jointly inform public evaluations of cultural-institution crises. Public comments are treated as sites where crisis meanings are formed, responsibility claims are advanced, and expectations for institutional response are expressed. This perspective provides the conceptual basis for understanding distributed accountability as the organization of functionally differentiated public demands across platform environments.

### Institutional legitimacy and crisis governance in cultural institutions

Cultural institutions gain legitimacy when their conduct is judged to be consistent with shared rules, professional norms, and public expectations [22,23]. Organizational legitimacy refers to a generalized perception that an entity’s actions are appropriate within a socially constructed system of norms, values, and beliefs [6]. Such legitimacy requires continuing maintenance as institutional pressures and public expectations change [24,25]. For museums and other cultural institutions, legitimacy depends on professional expertise, procedural propriety, governance transparency, custodial responsibility, and visible commitment to a public mission [5,8].

Institutional legitimacy becomes especially vulnerable during organizational crises, when controversies expose discrepancies between stated commitments and observed conduct [16,26]. In cultural institutions, these discrepancies may concern provenance ethics, procedural misconduct, governance failure, or neglect of public mission. They can prompt reassessments of professional authority and moral standing [27,28]. A discrete operational incident involving a misattributed object, a contested donation, or an unaccounted-for transfer can extend into wider judgments about an institution’s fitness as a custodial authority and its capacity to discharge public trust [7,15]. Museum legitimacy is shaped through evaluations within the professional field and among broader publics, including judgments of professional standards, perceived trustworthiness, and custodial credibility.

Changes in the communication environment have expanded the settings in which this vulnerability becomes visible. Public expectations increasingly emphasize transparency, responsiveness, and stakeholder inclusion [29,30]. The channels through which institutions are evaluated have also expanded. Platform discourse now operates alongside traditional media and professional evaluation as an additional setting in which institutional reputation is contested and renegotiated. Existing research on museum legitimacy has mainly examined institutional environments, governance structures, and internal processes of legitimacy maintenance and repair [7,15]. Less attention has been given to platform-mediated public judgment as a continuing source of external legitimacy pressure. Cultural institutions must therefore address both the precipitating incident and the public interpretations through which it becomes a test of custodial responsibility, procedural integrity, and institutional trust.

### Accountability in institutional crises

Accountability describes a relationship in which an actor is obliged to explain and justify conduct to a forum that can pose questions, render judgment, and impose consequences [31,32]. It operates as an ongoing process linking institutional actors to evaluative forums. Governance research distinguishes horizontal accountability among peer organizations, civil society groups, and media outlets from vertical accountability to hierarchical authorities [33]. Several accountability types may operate concurrently. Democratic accountability concerns responsiveness to public mandates. Professional accountability concerns adherence to expertise-based standards. Managerial accountability concerns the discharge of delegated responsibilities [34,35]. Cultural institutions may face all of these forms. Museums answer to professional communities on curatorial integrity, to funders and governing bodies on stewardship, and to broader publics on mission and trust [15,16].

Situational Crisis Communication Theory identifies perceived crisis responsibility as a central determinant of reputational threat and organizational response strategy [36], while related research links responsibility attribution to affective and behavioral responses [13]. Responsibility judgments vary with perceived controllability, severity, organizational history, and response strategy [12,14,37]. Higher perceived responsibility is associated with stronger expectations for accommodation and apology. Lower perceived responsibility is more commonly associated with explanatory or defensive response strategies [38,39]. Responsibility attribution therefore contributes to the formation and intensity of public accountability pressure and can connect discrete incidents with broader judgments about institutional character.

Platform environments alter how responsibility claims and accountability demands become visible. Comment sections, reposting chains, hashtag aggregation, and unofficial narratives can make responsibility claims visible before formal institutional responses appear. Blame-oriented comments may also shape perceptions of responsibility even when accompanying news coverage remains comparatively neutral [9–11,40]. Public comment spaces do not constitute formal accountability forums in the strict institutional sense. They provide semi-public environments in which demands for explanation, judgment, and consequences are produced and circulated. Participation is fluid, visibility is partly shaped by platform distribution systems, and normative pressure can accumulate through aggregated user responses [1,4]. These conditions require institutions to consider public accountability pressure beyond bounded settings such as regulatory hearings, press conferences, and professional reviews.

Cultural institutions remain comparatively understudied in this combined literature. Crisis communication research has concentrated mainly on corporate, governmental, and emergency settings, with cultural and heritage organizations appearing less frequently [12,41,42]. Museum controversies may activate professional standards, custodial trust, public mission, and moral expectations concerning cultural stewardship. Responsibility claims may extend beyond a specific incident into judgments about procedure, governance capacity, and public mission. Responsibility attribution and accountability pressure are therefore treated here as overlapping public processes that can inform wider assessments of institutional legitimacy.

### Platformization and the production of public judgment

Platformization provides a framework for understanding how digital platforms have become infrastructural to public communication. It describes the extension of platform logics across the web and the growing dependence of content, services, and audiences on platform infrastructures [43]. Research has extended this perspective to cultural production, public communication, and information infrastructures [20,21,44]. Platforms capture user activity through data infrastructures, organize visibility through algorithmic curation, and channel expression through programmable interfaces. These processes create communicative environments that are continuous and asynchronous, with consequences for how public judgments form and circulate.

Social media platforms bring organizational statements, user comments, reposts, hashtags, and secondary interpretations into connected communicative processes. Crisis narratives are repeatedly interpreted through interaction, and publics participate in producing and stabilizing crisis meanings [1,2,45,46]. Large volumes of user-generated content can intensify crisis visibility, while negative emotions can circulate rapidly across communicative boundaries [3]. Platform discussion also enables meaning negotiation under conditions of uncertainty. Users construct provisional interpretive frames through commenting, debating, and retelling, and these frames can influence subsequent public evaluation [47,48]. Existing research has explained crisis diffusion and public response, with less attention to how affective expression, information seeking, responsibility attribution, and institutional critique become connected within comment spaces during cultural-institution controversies.

The connection between platformization and museum legitimacy explains why platform-mediated discourse creates distinctive pressures for cultural institutions. Museum legitimacy depends on sustained perceptions that institutional conduct meets professional, custodial, and public-mission expectations [5–7]. These perceptions were historically shaped through professional evaluation, editorially mediated news coverage, and direct encounters between institutions and visiting publics. Platformization changes these conditions. Platform distribution systems help shape which controversies and interpretations become visible, while conflict-oriented content may receive greater circulation than routine institutional communication [1,49]. Comment infrastructures allow evaluations to accumulate asynchronously and remain accessible after the precipitating event. Cross-platform circulation also places the same controversy within different expressive conventions and community contexts. Observed differences in platform discourse may reflect a combination of content form, interaction structure, community convention, distribution systems, and visibility or moderation practices. The analysis does not identify the independent causal effect of any single platform mechanism. Museum legitimacy is consequently contested within communicative conditions that remain only partly addressed in institutional theory and museum studies.

### Distributed accountability as a function-based and platform-mediated concept

Distributed accountability is used here in a function-based sense to analyze the public-side organization of accountability pressure in platformized institutional crises. Prior scholarship has used the term in the context of multilevel governance to describe accountability distributed across multiple actors and governance arrangements [50]. The present framework shifts the analytical object from the distribution of accountability relationships to the distribution of substantive public demands for institutional response. Accountability research defines accountability through relationships in which actors must explain and justify their conduct before evaluating forums [31,32]. Crisis communication research shows how responsibility attribution shapes anger, reputational threat, and expectations for organizational response [12–14]. Platformization adds communicative environments in which data infrastructures, algorithmic curation, interfaces, and community conventions influence the visibility and circulation of evaluative expression [20,21,43]. Distributed accountability refers to the concurrent production of functionally differentiated demands for institutional responsiveness across these environments.

Public judgment in an institutional crisis seldom takes the form of a single demand from a unified public. It may contain several accountability functions with different evaluative criteria and response requirements. Punitive accountability concerns culpability, investigation, sanctions, and visible consequences. Knowledge-based accountability concerns explanation, procedural reasoning, expertise, documentation, and comparative evidence. Together with ethical accountability as defined above, these functions draw on established distinctions among managerial, professional, and democratic accountability while directing analytical attention to the substantive demands expressed through public accountability pressure in platformized crisis settings [34,35].

Platform environments may influence the relative visibility and prevalence of these functions through content distribution, interface affordances, content formats, and community conventions [1,9,10]. The functions remain analytically independent of any particular platform. Each function can appear in any platform corpus, and one corpus may contain a mixed profile across several functions. The framework does not assume that one function must predominate in every environment. Distributed accountability therefore describes the functional disaggregation of public judgment under platformized communication. It identifies concurrent demands generated around one institutional crisis and permits comparison of their relative salience across communicative environments. Platform environments are treated as mediating conditions, not as constitutive sources of particular accountability functions. Accordingly, differences in observed function distributions are interpreted as contingent associations within platform-specific corpora, not as evidence that platform architecture determines the form of accountability expressed by users.

Distributed accountability differs from networked accountability in its analytical object. Networked accountability examines relations among actors, forums, and oversight arrangements [33,35]. It identifies who is expected to answer to whom and how accountability relationships are organized. Distributed accountability distinguishes the substantive demands directed at an institutional actor and examines their distribution across communicative environments. A relational map can identify accountability connections without specifying whether a public demand seeks sanction, evidentiary explanation, or relational repair.

This distinction provides a different analytical focus. Relational approaches identify accountability connections among actors and forums but do not necessarily classify the substantive response demanded in each connection. Distributed accountability separates demands according to their response requirements because sanctions, evidentiary explanations, and acts of relational repair are not interchangeable. The framework also permits comparison of the relative salience of these demands across platforms and controversies. Mixed functional profiles and the absence of one-to-one platform-function correspondence can therefore be treated as empirical patterns. The concept concerns accountability pressure and public demand. It does not describe realized accountability outcomes or establish that public demands produce institutional consequences.

### Analytical framework

The preceding literature supports a two-dimensional framework for examining public judgment during cultural-institution crises. One dimension comprises affective expression, responsibility attribution, and legitimacy questioning. These layers provide conceptual categories for interpreting different forms of evaluative activity in public comments. The other dimension comprises punitive, knowledge-based, and ethical accountability. These functions classify the forms of institutional response sought through public demands. The layers and functions intersect, but they do not correspond one to one. The two dimensions differ in analytical status in this study. The three accountability functions are operationalized through the thematic-group-to-function mapping described in the Methods. The three layers are used as an interpretive reading of topic content and are not separately coded or counted.

The relative salience of the thematic groupings and accountability functions is compared across event-anchored content groups and platform-specific corpora. Platform environments are treated as conditions that may influence the visibility and distribution of accountability functions. Fig 1 presents these relationships without assigning any function to a particular platform.

**Fig 1:**
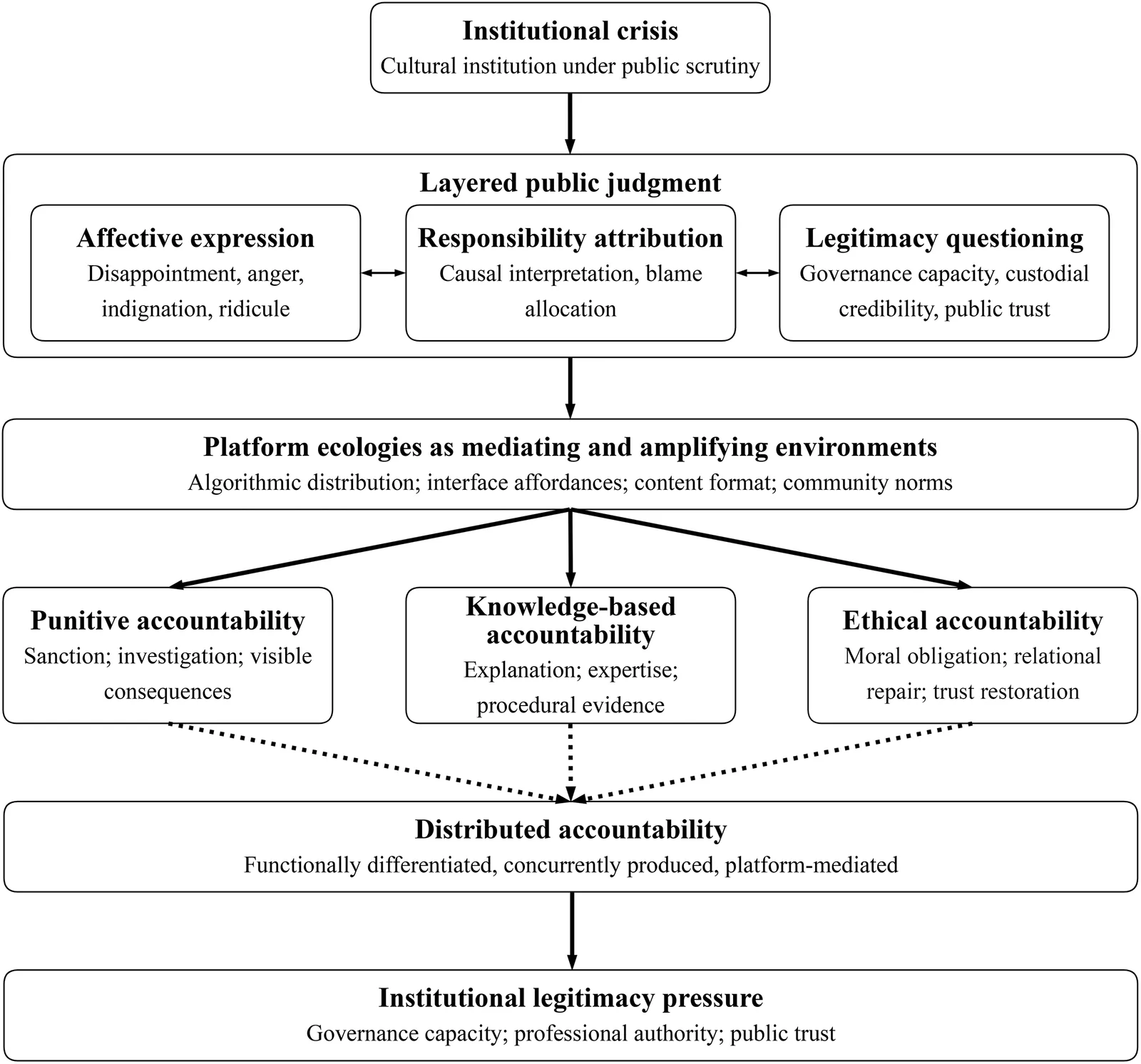
Conceptual framework of distributed accountability in a platformized institutional crisis. The framework distinguishes three overlapping layers of public judgment, comprising affective expression, responsibility attribution, and legitimacy questioning. These layers are conceptual and were not measured in the present analysis. Accountability demands are classified into punitive, knowledge-based, and ethical functions. The layers describe forms of public evaluation, while the functions describe the institutional responses sought through those evaluations. These dimensions may intersect without forming one-to-one relationships. Platform environments are treated as mediating conditions that may shape the relative salience of each function, while every function can appear in any platform corpus. Distributed accountability refers to the concurrent presence and uneven distribution of these functions across communicative environments. The framework does not assign a single accountability function to a specific platform or claim that public demands necessarily produce institutional consequences. Vertical arrows indicate analytical relationships within the framework and do not represent an estimated causal sequence.

## Materials and methods

### Research design

This study examines the Nanjing Museum donated-artifact controversy to analyze how public accountability demands are organized across platform environments. The design compares two dimensions. One is the platform environment in which comments appeared. The other is the event-anchored content group of the source content to which comments were attached. Computational topic modeling was used to identify first-order topics from the comment corpus. Higher-order thematic groupings were developed through an initial round of independent researcher coding followed by consensus discussion. The resulting coding framework was then fixed and applied in a second independent coding round to assess the reproducibility of topic-level classification. Platform-specific corpora are treated as differentiated empirical environments for public judgment, consistent with the function-based and platform-mediated framework developed above.

### Case background and case selection

The Nanjing Museum is one of China’s earliest modern museums and a major comprehensive museum in China. Established in 1933 as the Preparatory Office of the National Central Museum, it now operates as a national first-class museum under the Jiangsu Provincial Department of Culture and Tourism, with collections exceeding 430,000 items and one of the country’s largest holdings of designated precious cultural relics. The Pang family donation at the center of the controversy derives from the collection assembled by Pang Yuanji (1864–1949, also known as Pang Laichen, studio name Xuzhai), a prominent collector of Chinese painting during the late Qing and Republican periods. His Xuzhai collection is now distributed primarily among the Palace Museum, the Shanghai Museum, the Nanjing Museum, and the Suzhou Museum. In 1959, Pang Zenghe, the collector’s grandson, made an uncompensated donation of 137 paintings from the Xuzhai collection to the Nanjing Museum, an act formally recognized by the Jiangsu Provincial People’s Council in 1962. The sequence of key public developments from December 2025 to the release of the official findings in February 2026 is summarized in the Introduction and is not repeated here. Four features informed the selection of this case. The institution holds significant national standing, which raises the stakes attached to custodial and procedural conduct. The controversy involves an institutional history reaching back six decades, which allows questions of documentation and governance continuity to become publicly salient. The dispute concerns custodial and donor-relational obligations that are central to museum legitimacy. Public discussion developed concurrently on platforms with contrasting content forms and comment environments, which permits comparison across communicative settings within one controversy.

The corpus is organized into three event-anchored content groups defined by the event phase of the selected source content. Node A comprises comments attached to source content associated with the museum’s initial statement and the National Cultural Heritage Administration’s intervention between 17 and 23 December 2025. Node B comprises comments attached to source content associated with the continuing joint investigation and related developments between 27 and 30 December 2025. Node C comprises comments attached to source content associated with the release of the official investigation findings, the museum’s public apology, and subsequent discussion between 9 and 27 February 2026. These date ranges define the event phases associated with the selected source content. They do not define the posting dates of the comments themselves. Node membership was assigned according to the original collection package of each source item, and comments inherited the Node assigned to their source content. The date ranges attached to Nodes A, B, and C identify the event focus of the corresponding source-content collection packages; they were not used as publication-date eligibility windows for individual source items or as posting-time bins for comments. Comment posting dates therefore overlap across groups, and some comments may predate the event dates associated with the collection package to which their source item was assigned. Comparisons across groups describe differences among event-anchored source-content collections and are not interpreted as a temporal progression of public opinion.

### Platform selection and data collection

The study focuses on Douyin, Bilibili, and Xiaohongshu (Rednote), which represent three structurally different content environments within the Chinese platform ecosystem. Douyin is a Chinese mobile short-video platform organized around personalized feed-based content distribution [51]. Bilibili is a video-sharing platform distinguished by its danmu (bullet-comment) interface alongside conventional comment sections; research has linked these practices to participatory community formation, while its user base is characterized primarily by adolescents and young adults [52,53]. Rednote is a Chinese user-generated content platform centered on text-and-image sharing, search, personalized recommendation, and lifestyle- and consumption-oriented information exchange, with a predominantly young and female user base [54]. These differences in content format, interaction structure, user composition, and established participation practices provide the comparative basis for examining whether the same institutional controversy is organized differently across platform-specific comment corpora.

During preliminary scoping, Weibo, WeChat, and Kuaishou were also examined. Case-related discussion on these platforms was less extensive or less consistently retrievable during repeated screening. The final corpus therefore focused on Douyin, Bilibili, and Rednote, which provided sufficiently substantial and comparable public comment environments for the study.

Candidate source content was identified manually on each platform using event-specific Chinese-language search terms entered in Chinese characters under the platforms’ default search-ranking settings. For readability, representative search combinations are reported below in Hanyu Pinyin with English glosses. Newly registered ordinary user accounts were used for the searches. The same event-specific search logic was applied across Douyin, Bilibili, and Rednote. The search terms served as discovery anchors for locating potentially relevant source content and were not used as an automated inclusion rule. For Node A, searches used combinations of terms relating to the museum’s initial response, donated paintings and calligraphy, their whereabouts, authenticity, and earlier disposal. Representative search combinations included “*Nanjing Bowuyuan qingkuang shuoming juanzeng shuhua*” (Nanjing Museum statement on donated paintings and calligraphy), “*Nanbo shengming juanzeng shuhua quxiang*” (Nanjing Museum statement on the whereabouts of donated paintings and calligraphy), “*Nanjing Bowuyuan juanzeng shuhua weizuo chuzhi*” (Nanjing Museum disposal of donated paintings and calligraphy classified as forgeries), and “*Nanjing Bowuyuan huiying juanzeng shuhua*” (Nanjing Museum response concerning donated paintings and calligraphy). For Node B, searches used combinations of terms relating to the subsequent investigation and the involvement of cultural-heritage authorities and investigation teams. Representative search combinations included “*Nanjing Bowuyuan diaochazu jieru diaocha*” (investigation team intervention in the Nanjing Museum case), “*Nanjing Bowuyuan quanmian diaocha tongbao*” (announcement concerning the comprehensive investigation of the Nanjing Museum case), “*Wenwuju gongzuozu Nanjing Bowuyuan hecha*” (cultural-heritage authority working group verification of the Nanjing Museum case), and “*Jiangsu diaochazu Nanjing Bowuyuan*” (Jiangsu investigation team and the Nanjing Museum case). For Node C, searches centered on combinations of terms relating to the final investigation findings and official announcement. These included “*Nanjing Bowuyuan*” (Nanjing Museum), “*tongbao*” (official announcement), “*diaocha jieguo*” (investigation findings), “*chuli jieguo*” (handling or disciplinary outcomes), and related terms referring to the February 2026 official findings and subsequent institutional response. These terms were entered in multiple related combinations during manual platform searches. No single indivisible query string was used. Search results were then reviewed manually. Substantive relevance to the corresponding event node served as the primary eligibility criterion. Among relevant candidate source items, visible engagement indicators, particularly like and comment counts, were considered as additional selection criteria so that the retained materials contained sufficient public discussion for comment-level analysis. Source selection was therefore purposive and did not consist of automatically retaining the first or highest-ranked search results. Fifty source entries were ultimately retained for each platform-by-node combination, yielding a final sampling frame of 450 source entries. The retained source items were then recorded as platform-specific source identifiers or URLs and supplied to the corresponding collection procedures for comment retrieval.

Publicly visible comments associated with the retained source items were collected using platform-specific collection procedures. Retrieval generally yielded on the order of 200 raw comment records per source, although the number varied across source items and platforms and some sources yielded more than 200 top-level comments. The preserved collection materials do not document a single hard cross-platform comment cap or an identical pagination rule. Raw retrievals could include nested replies; these were removed during preprocessing so that the final analytic corpus contained only publicly visible top-level comments. Data collection was conducted using a Python-based collection tool through ordinary registered platform accounts, with low-frequency sequential requests and local structured-data storage. Collection was limited to content available through ordinary platform access; no private or restricted-access content was retrieved, and no access controls were circumvented. The authors confirm that the collection and analysis procedures complied with the terms and conditions applicable to the source platforms at the time of data collection. The research team first accessed platform data for research purposes on 8 February 2026, and data collection was completed on 12 March 2026. During collection, publicly visible account-level identifiers associated with comments were technically accessible through the platform data structure, but these identifiers were excluded from the analytic dataset and were not retained for analysis or public release.

Core analytic fields included comment text, posting time, platform source, and source-content identifier. Because platform data structures differ, the corpus was standardized during preprocessing to support comparison. Across the source files, 88,552 comment observations were initially available. Comment-level deduplication based on platform and comment identifier removed 13,097 duplicate observations. A further 406 records with empty text and 154 records with posting timestamps beyond the predefined platform-specific analytical cutoffs were excluded, followed by the removal of 10,546 nested replies. The resulting locked modeling corpus comprised 64,349 publicly visible top-level comments across the three platforms and three event nodes, with comment volumes reported in Table 1.

**Table 1:** Composition of the analytic corpus by platform and event node.

Platform	Node A (Dec 17–23)	Node B (Dec 27–30)	Node C (Feb 9–27)	Total
Douyin	9,895	9,950	8,301	28,146
Bilibili	8,000	7,288	2,031	17,319
Rednote	7,547	7,546	3,791	18,884
Total	25,442	24,784	14,123	64,349

Nodes are defined by the event phase of the selected source content. Node assignment does not follow comment posting date. Counts represent the locked top-level-comment corpus after comment-level deduplication, exclusion of empty-text records and comments posted beyond the predefined platform-specific analytical cutoffs, and removal of nested replies.

Comment posting and data-collection windows were retained separately because Node assignment was based on source-content event groups. Comment posting dates did not determine node assignment. Comments in the locked corpus were posted between 17 December 2025 and 12 March 2026. Posting and collection windows by platform and event node are reported in Table 2.

**Table 2:** Comment posting and data collection windows by platform and event node.

Platform	Node	Comment posting	Data collection
Douyin	A	17/12/2025 to 09/02/2026	08/02/2026 to 09/02/2026
Douyin	B	23/12/2025 to 27/02/2026	27/02/2026
Douyin	C	09/02/2026 to 10/03/2026	08/03/2026 to 11/03/2026
Bilibili	A	17/12/2025 to 25/02/2026	27/02/2026
Bilibili	B	23/12/2025 to 25/02/2026	27/02/2026
Bilibili	C	09/02/2026 to 12/03/2026	12/03/2026
Rednote	A	17/12/2025 to 23/02/2026	27/02/2026
Rednote	B	17/12/2025 to 27/02/2026	27/02/2026
Rednote	C	09/02/2026 to 11/03/2026	11/03/2026

Nodes are assigned according to the event phase of the selected source content. Comment posting date does not determine node assignment. Comment posting windows report the observed posting dates of comments in the locked corpus. Data-collection windows report the corresponding collection period. Single dates indicate that collection for the corresponding platform–node group was completed on that date.

### Preprocessing and topic modeling

Corpus construction and embedding preparation were treated as separate stages. Comment-level duplicate observations had already been removed during corpus construction. For semantic embedding, the retained raw comment text was passed directly to the pretrained Chinese embedding model M3E-base without global text deduplication, entity normalization, stopword removal, or word segmentation [55]. BERTopic [56] was applied for unsupervised topic identification, combining UMAP for dimensionality reduction [57], HDBSCAN for density-based clustering [58], and Maximal Marginal Relevance for keyword diversification [59].

For topic representation, Chinese tokenization used jieba.lcut with the default Jieba dictionary. CountVectorizer was configured with token_pattern = None, lowercase = False, ngram_range=(1, 1), min_df = 10, and max_df = .85 during model fitting. Topic representations were subsequently refined using c-TF-IDF and lexical Maximal Marginal Relevance (MMR; diversity = .30), with unigram and bigram candidates generated using ngram_range = (1, 2), a 122-item Chinese stopword list, and lexical filtering of non-substantive tokens. These representation-stage procedures affected the displayed topic terms. The raw texts used to generate semantic embeddings were unchanged. UMAP was configured with 25 nearest neighbors, five dimensions, a minimum distance of 0.0, cosine distance, and a fixed random state of 42. HDBSCAN used a minimum cluster size of 80, a minimum sample size of 10, Euclidean distance, and the excess-of-mass cluster-selection method. Outlier reassignment used a probability-based strategy with a threshold of 0.10, and topic reduction targeted a final solution of 50 topics. The random seed was fixed at 42. A targeted parameter-sensitivity analysis examined the document-level clustering structure underlying BERTopic by varying the UMAP neighborhood size (n_neighbors = 15 and 35) and the HDBSCAN minimum cluster size (min_cluster_size = 60 and 100) around the baseline specification while holding the embedding model and other clustering parameters constant. Stability was assessed using the adjusted Rand index (ARI), adjusted mutual information (AMI), changes in raw HDBSCAN outlier rates, and ARI among documents classified as non-outliers in both the baseline and alternative specifications. The sensitivity analysis evaluates the UMAP–HDBSCAN clustering stage before final topic reduction. It assesses the stability of the underlying clustering structure and does not directly assess the stability of the reduced 49-topic solution. The full preprocessing, modeling, software environment, and data availability protocol are provided in S2 File. The analysis code, deidentified analytical data, and reproducibility materials supporting the reported downstream analyses are provided in S3 File.

### Thematic consolidation and comparative analysis

Higher-order thematic coding followed a two-stage procedure that combined computational topic discovery with structured researcher interpretation and validation, consistent with established methodological approaches to the qualitative interpretation and validation of computational text models [60,61]. In the initial framework-development stage, two coders independently reviewed the topic-level materials without access to each other’s assignments and developed provisional higher-order classifications from the semantic content of the BERTopic topics. They subsequently compared their independent classifications and resolved differences through discussion. This process produced a consensus higher-order framework comprising six substantive thematic groupings, together with a mixed category (M) and an insufficiently interpretable or low-information category (X). The agreed framework was then formalized as a written codebook containing a core definition, inclusion indicators, and explicit boundary rules for each category. The framework was fixed before the subsequent independent validation round.

In the subsequent validation stage, the two coders independently classified all 49 substantive topics in the final BERTopic solution using the fixed written codebook. Both coders received the same blinded topic-level materials, including topic size, ranked topic-representation terms, and deidentified representative comments, and neither had access to the other coder’s validation assignments or the later adjudicated mapping. No thematic category was added, removed, or redefined during the validation round. Intercoder reliability was evaluated at this fixed-framework stage, consistent with established guidance for codebook-based qualitative coding [62]. In the independent validation round, the coders agreed on 35 of 49 topic assignments, corresponding to 71.43% raw agreement and Cohen’s κ = .66 (95% bootstrap CI [.50,  .80]; 10,000 bootstrap resamples, random seed = 42). Cohen’s κ therefore quantifies the reproducibility with which the two coders applied the fixed higher-order framework to the 49 topic-level units; it does not quantify the earlier consensus process through which the framework was developed.

The consolidation grouped first-order topics for reporting and comparison without changing comment-level topic assignments. The finalized coding framework also linked the six substantive thematic groupings to three accountability functions: G1 and G4 to ethical accountability, G2 and G5 to knowledge-based accountability, and G3 and G6 to punitive accountability. M and X were not assigned to an accountability function. Coders assigned topics at the thematic-group level; accountability functions were not coded as a separate judgment and followed from the assigned thematic-group code. Applying this fixed mapping to the two frozen independent validation assignments yielded 77.55% function-level agreement across all 49 topics (38/49; Cohen’s κ = .692). Among the 41 topics for which both coders independently assigned one of G1–G6, function-level agreement was 80.49% (33/41; κ = .706). Because the function-level results depend on the topic-to-group assignments, sensitivity analyses were conducted using the frozen independent validation assignments of Coder A and Coder B and a specification in which Topic 0, the largest disputed topic, was treated as mixed and excluded from function-level analysis. For RQ1 and RQ2, an additional sensitivity analysis compared the primary consensus specification with the Topic 0 exclusion specification. These analyses retained the BERTopic solution and all other topic assignments unchanged. The primary specification retains the consensus assignments that had been locked before the sensitivity analyses were conducted. Retaining this specification avoids redefining the primary coding after observing the consequences of alternative treatments; accordingly, absolute prevalence rankings that vary across specifications are not interpreted as stable findings.

The three layers of public judgment set out in the analytical framework were not operationalized as coded variables. No sentiment, emotion, or attribution measurement was applied at comment level. All frequencies, tests, and effect sizes reported below refer to the six thematic groupings and the three accountability functions. For RQ1, topic-level and higher-order thematic distributions were described across all 42,126 comments assigned to the 49 substantive topics. For RQ2, the relative salience of the higher-order thematic groupings was compared across event nodes using within-node percentages calculated from the substantive-topic corpus. M and X were retained separately and were not reassigned to the six substantive groups. For RQ3, the three accountability functions were compared across the 41,383 comments assigned to G1–G6. Platform differences in accountability-function distributions were assessed using a chi-square test of independence, Cramér’s *V*, and adjusted standardized residuals. These statistics are interpreted descriptively as properties of the analyzed corpora. They are not causal estimates of platform effects.

### Ethical considerations

The study analyzed publicly visible comments accessed through ordinary registered platform accounts. No private messages or restricted-access materials were collected, and the collection process did not circumvent platform access controls. During collection, some account-level metadata associated with publicly visible comments were technically accessible as part of the platform data structure. Direct account identifiers were not included in the analytic dataset or in the public supporting files. Identifying platform metadata and traceable comment-level identifiers were excluded from all public supporting materials, and results are reported only in aggregated or topic-level form. No usernames, profile identifiers, comment identifiers, source-content identifiers, URLs, or other directly traceable identifiers are reported in the manuscript or public supporting files. The principal privacy risk is that verbatim public comments may be traceable to individual platform users through search or platform retrieval. To reduce this risk, directly traceable identifiers were excluded from analytic and public outputs, and representative expressions reported in the manuscript were translated or paraphrased. The study examines public judgment and accountability pressure and does not adjudicate the legal or factual responsibility of any individual actor. Ethical approval for this study was granted on 9 January 2026 by the Chair’s Action of the Faculty Ethics Committee, Faculty of Humanities and Social Sciences, City University of Macau (Approval No. FHSS250233). The approved ethics application included a request for waiver of written informed consent for this non-interventional study of publicly visible online comments. No individual informed consent was obtained, and the study involved no direct interaction with platform users.

## Results

### Sample overview and topic model output

The source files contained 88,552 comment observations. After comment-level deduplication, exclusion of empty-text and post-cutoff records, and removal of nested replies, the locked modeling corpus contained 64,349 top-level comments, including 28,146 from Douyin, 17,319 from Bilibili, and 18,884 from Rednote. Across event nodes, 25,442 comments were assigned to Node A, 24,784 to Node B, and 14,123 to Node C. The initial clustering solution contained 149 substantive topics and one outlier label. A total of 23,593 comments (36.66%) were initially classified as outliers. Probability-based reassignment assigned 1,370 of these comments to substantive topics, leaving 22,223 final outliers (34.54%). After topic reduction, the final BERTopic solution contained 49 substantive topics and one outlier cluster. The 49 substantive topics comprised 42,126 comments and formed the basis of the topic-level analyses. The targeted sensitivity analysis showed high agreement among documents classified as non-outliers in both the baseline and alternative specifications (common-nonoutlier ARI = .934 to  .951). Agreement across all documents ranged from ARI = .564 to  .879 and AMI = .787 to  .949, while raw HDBSCAN outlier rates ranged from 33.63% to 37.17%. These results indicate that the clustering structure among consistently retained documents was comparatively stable. Outlier assignment was more sensitive to parameter variation. As specified in the Methods, the sensitivity analysis evaluates the UMAP–HDBSCAN clustering stage. It does not evaluate the final reduced 49-topic solution.

### Overall issue structure and thematic groupings

The two-stage coding procedure produced a fixed six-group higher-order thematic framework, which was applied to the final 49 substantive topics in the independent validation round. The six groupings comprise donation ethics and restitution, authenticity disputes and circulation, investigation and punitive accountability, trust erosion and legitimacy, historical comparison and media circulation, and personalized blame and family implication. In the validation round, independent coding of the 49 substantive topics produced 71.43% raw agreement (35/49) and Cohen’s κ = .66 (95% bootstrap CI [.50,  .80]). The 14 validation-round disagreements were resolved through discussion and final consensus review. Following adjudication, 44 topics were assigned to the six substantive thematic groupings. One topic containing 88 comments was retained as mixed (M), and four topics containing 655 comments were classified as insufficiently interpretable or low-information (X). The six substantive groupings therefore contained 41,383 comments, representing 98.24% of the 42,126-comment substantive-topic corpus. The final coding assignments are reported in Table 3.

**Table 3:** Final BERTopic topics, thematic assignments, top keywords, and proportions.

Thematic grouping	Topic	Top keywords	Proportion
Donation ethics and restitution	2	donation; donor; restitution	0.0799
	4	Pang family; descendants; donation	0.0479
	9	unresolved claims; restitution; recovery	0.0295
Authenticity disputes and circulation	3	authentication; auction; forgery	0.0593
	8	counterfeit; auction price; forgery	0.0304
	22	invoice; purchaser; provenance	0.0084
	24	expert; authentication; appraisal	0.0079
	25	national treasure; loss; recovery	0.0076
	26	item count; discrepancy; provenance	0.0071
	47	auction; painting price; provenance	0.0020
Investigation and punitive accountability	1	museum; cultural relics; Nanjing Museum	0.1202
	5	reporting; police; crime	0.0420
	7	investigation; cross-regional probe; accountability	0.0373
	10	death penalty; execution; punishment	0.0289
	21	discipline inspection; corruption; intervention	0.0092
	23	apology; legal accountability; restitution	0.0084
	27	insider theft; dereliction of duty; punishment	0.0065
	38	corrupt official; embezzlement; punishment	0.0030
	46	insider theft; prevention; family	0.0021
Trust erosion and legitimacy	0	state institutions; auction; public distrust	0.2066
	6	Nanjing Museum; credibility; cultural relics	0.0376
	13	ridicule; mockery; absurdity	0.0207
	14	local government; Nanjing; Jiangsu	0.0181
	17	cover-up; fire; warehouse	0.0146
	31	cockroach; hidden corruption; metaphor	0.0035
	34	darkness; cover-up; corruption	0.0032
	35	delayed justice; absent justice; legitimacy	0.0031
	40	rotten to the core; uprooting; corruption	0.0028
	43	collective; individual; institutional	0.0022
	44	symbolic punishment; leniency; token penalty	0.0022
Historical comparison and media circulation	11	Palace Museum; Taipei; historical comparison	0.0221
	15	nine clans; historical punishment; comparison	0.0165
	18	tomb raiding; cultural relics; comparison	0.0108
	29	Guangming Daily; media coverage; historical comparison	0.0057
	30	spectator; gossip; media	0.0054
	39	appraisal livestream; antiques; media	0.0030
	41	beheading; historical comparison; punishment	0.0024
	42	commentator; narrative; media	0.0023
	48	Asia Weekly; media; banned	0.0019
Personalized blame and family implication	12	Director Xu; family implication; personal blame	0.0220
	16	museum director; suicide; personal blame	0.0153
	19	elderly individual; personal blame; family	0.0104
	20	family lineage; generational blame; son	0.0095
	37	family confiscation; clan punishment; personal blame	0.0030
Mixed (M)	45	mixed; ambiguous; unclear	0.0021
Insufficiently interpretable or low-information (X)	28	acknowledgment; support; uninterpretable	0.0060
	32	belief; vague; uninterpretable	0.0034
	33	support; investigation; uninterpretable	0.0032
	36	golden hoop; museum; uninterpretable	0.0030

Proportions represent each topic’s share of the 42,126 comments assigned to the 49 substantive BERTopic topics. Topics coded as mixed (M) or insufficiently interpretable/low-information (X) were retained in topic-level reporting but were not mapped onto the six substantive thematic groupings or the three accountability functions. Proportions may not sum to 1.00 because of rounding.

Under the primary consensus coding, trust erosion and legitimacy accounted for the largest share of substantive-topic comments (13,251 comments, 31.46%), followed by investigation and punitive accountability (10,843, 25.74%). Donation ethics and restitution accounted for 15.72%, authenticity disputes and circulation for 12.28%, historical comparison and media circulation for 7.01%, and personalized blame and family implication for 6.03%. The remaining 1.76% of comments belonged to mixed or insufficiently interpretable topics and were retained outside the six-group comparative framework. The absolute ranking of the thematic groupings was sensitive to the treatment of Topic 0, the largest final topic and one of the intercoder disagreements. When Topic 0 was treated as mixed and excluded from the six substantive groupings, G4 decreased from 13,251 to 4,547 comments (10.79% of the 42,126-comment substantive-topic corpus), and investigation and punitive accountability became the largest substantive grouping. The relative ordering of G4 should therefore not be interpreted as a stable prevalence finding. These results show that public discussion extended beyond questions about the status and authenticity of individual works. Institutional trust, responsibility attribution, donor obligations, historical comparison, and personalized blame formed an interconnected structure of public judgment.

The six groupings can be interpreted through the three overlapping layers of public judgment proposed in the analytical framework. An affective reading is supported by topic-representation terms foregrounding ridicule, mockery, absurdity, and spectatorship, including Topics 13 and 30. Responsibility-oriented content is visible in topics organized around reporting, investigation, accountability, and personalized blame, including Topics 5, 7, and 12. Legitimacy-oriented content is reflected in topics organized around public distrust, perceived cover-up, and delayed justice, including Topics 0, 17, and 35. These examples illustrate how the three-layer framework can be used to interpret the topic solution. They do not constitute a separate classification of topics or comments into the three layers, and the study does not estimate the prevalence or overlap of these layers.

### Differences across event nodes

Comparison across event-anchored source-content groups showed clear differences in the relative composition of the six substantive thematic groupings. Under the primary consensus coding, trust erosion and legitimacy accounted for the largest share in Nodes A and B. The investigation and punitive accountability grouping was narrowly the largest in Node C.

In Node A, trust erosion and legitimacy accounted for 29.34% of substantive-topic comments, while donation ethics and restitution accounted for 22.12% and investigation and punitive accountability for 20.66%. Donation ethics was therefore considerably more prominent in Node A than in Node B (9.70%) or Node C (13.68%). This pattern is consistent with the stronger salience of donor obligations, restitution, and the implications of institutional handling of donated works in discussion attached to the initial response phase.

Node B contained a greater concentration of investigation-oriented discussion. Investigation and punitive accountability accounted for 28.26% of comments, while trust erosion and legitimacy accounted for 34.67% under the primary consensus coding. Personalized blame and family implication also increased to 7.99%, compared with 3.18% in Node A.

In Node C, investigation and punitive accountability accounted for 31.27% of substantive-topic comments, narrowly exceeding trust erosion and legitimacy at 30.04%. Personalized blame and family implication accounted for 8.18%, while donation ethics and restitution accounted for 13.68%. Discussion attached to the official findings and subsequent response therefore continued to combine institutional distrust with responsibility attribution and increasingly personalized forms of blame.

The node comparison shows that all six thematic groupings were present in every event-anchored discourse setting, with their relative salience differing among nodes. Comments attached to source content from different nodes overlap in posting dates. These differences are therefore interpreted as contrasts among event-anchored discourse settings. They do not describe a temporal progression of public opinion. Fig 2 presents the within-node distribution of the six substantive thematic groupings. The absolute prominence of G4 across nodes was sensitive to the treatment of Topic 0. Under the primary consensus coding, G4 was the largest grouping in Nodes A and B, but after treating Topic 0 as mixed, G1 became the largest grouping in Node A and G3 became the largest in Nodes B and C. The principal between-node contrasts were unchanged. G1 remained relatively higher in Node A, and G3 and G6 remained relatively higher in Nodes B and C. The Node × G1–G6 association remained significant under both specifications. Under the primary consensus coding, χ²(10, *N* = 41,383) = 2454.00, *p* < .001, Cramér’s *V* = .172. When Topic 0 was treated as mixed and excluded from the six substantive groupings, χ²(10, *N* = 32,679) = 2362.63, *p* < .001, Cramér’s *V* = .190.

**Fig 2:**
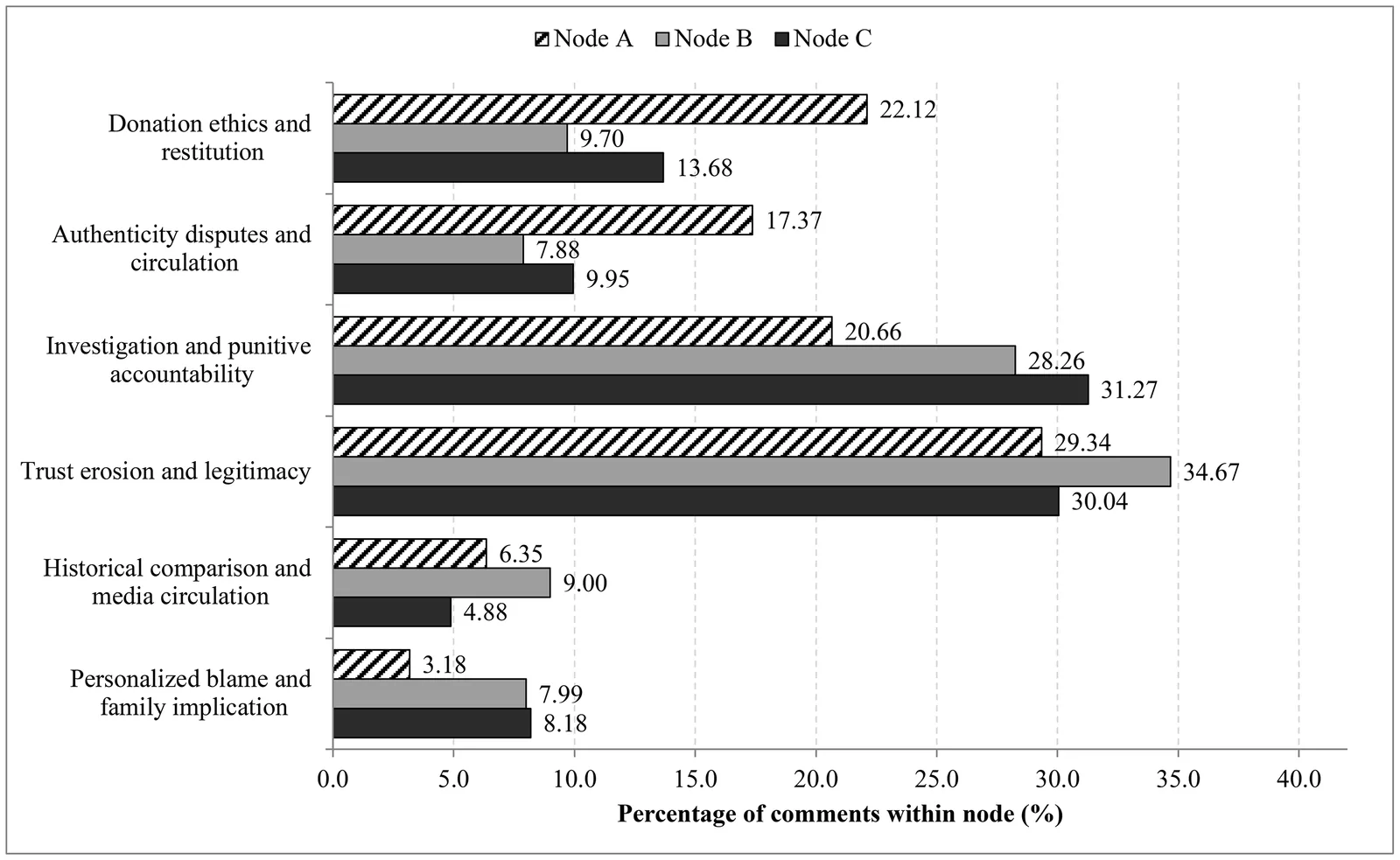
Distribution of higher-order thematic groupings across event nodes. The figure shows within-node percentages for the six substantive thematic groupings across Nodes A, B, and C. Nodes are defined by the event phase of the selected source content. Node assignment does not follow comment posting date. Mixed and insufficiently interpretable topics are not displayed. Because M and X were retained in the within-node denominator but are not displayed, the six percentages do not necessarily sum to 100%. Percentages shown are based on the primary consensus coding; coding-sensitivity results are reported in S1 File.

### Cross-platform differences in accountability structures

Under the primary consensus coding, ethical accountability accounted for the largest share of the 41,383 comments assigned to the six substantive thematic groupings (19,875 comments, 48.03%), followed by punitive accountability (13,382, 32.34%) and knowledge-based accountability (8,126, 19.64%). All three functions were present in each platform corpus, but their relative distributions differed across platforms. The association between platform and accountability function was statistically significant, χ²(4, *N* = 41,383) = 343.43, *p* < .001, although the overall association was small (Cramér’s *V* = .064).

Under the primary consensus coding, ethical accountability accounted for 45.51% of classified Douyin comments, punitive accountability for 36.84%, and knowledge-based accountability for 17.65%. Relative to the pooled distribution under this specification, punitive accountability was strongly over-represented (adjusted standardized residual = 17.55). Knowledge-based (−9.12) and ethical accountability (−9.18) were under-represented. Douyin therefore showed the strongest relative punitive orientation of the three platform corpora. This relative punitive orientation remained stable across the alternative coding specifications examined below.

Under the primary consensus coding, ethical accountability accounted for 50.33% of classified Bilibili comments, followed by punitive accountability (27.31%) and knowledge-based accountability (22.36%). Relative to the pooled distribution under this specification, punitive accountability was under-represented (adjusted standardized residual = −12.66), while knowledge-based accountability (+8.09) and ethical accountability (+5.42) were over-represented. Bilibili therefore showed a clear relative concentration of knowledge-based accountability. This relative knowledge-based orientation remained stable across the alternative coding specifications examined below.

Under the primary consensus coding, ethical accountability accounted for 49.81% of classified Rednote comments, punitive accountability for 29.89%, and knowledge-based accountability for 20.30%. Relative to the pooled distribution under this specification, punitive accountability was under-represented (adjusted standardized residual = −7.03), while knowledge-based (+2.23) and ethical accountability (+4.80) were above expectation. Rednote showed smaller deviations from the pooled distribution than Douyin and did not display a comparably strong punitive or knowledge-based concentration.

Across platforms, all three accountability functions were present in different proportions. Under the primary consensus coding, ethical accountability accounted for the largest share in each platform corpus, but the sensitivity analysis below shows that this absolute ordering is not stable across coding specifications. The more stable cross-platform pattern concerns relative composition. Douyin showed a comparatively stronger punitive profile, Bilibili showed a comparatively stronger knowledge-based profile, and Rednote showed smaller departures from the pooled distribution. The platform–function association was statistically significant but small. The absolute ranking of the three accountability functions was sensitive to coding specification. Ethical accountability was largest under the primary consensus specification and the Coder B specification. Punitive accountability was largest under the Coder A specification and when Topic 0 was excluded. The platform–function association remained statistically significant across all four specifications, with Cramér’s *V* ranging from .052 to  .070. The relative cross-platform pattern was stable. Douyin consistently showed a relative punitive concentration, and Bilibili consistently showed a relative knowledge-based concentration. These sensitivity results indicate that the absolute prevalence ranking of accountability functions depends on the treatment of Topic 0. Table 4 reports the observed platform-by-function distributions, within-platform percentages, and adjusted standardized residuals.

**Table 4:** Cross-platform comparison of accountability structures in the Nanjing Museum controversy.

Platform	Punitive accountability, *n* (%)	Adj. residual	Knowledge-based accountability, *n* (%)	Adj. residual	Ethical accountability, *n* (%)	Adj. residual	Total
Douyin	6,787 (36.84%)	17.55	3,251 (17.65%)	−9.12	8,384 (45.51%)	−9.18	18,422
Bilibili	2,838 (27.31%)	−12.66	2,324 (22.36%)	8.09	5,230 (50.33%)	5.42	10,392
Rednote	3,757 (29.89%)	−7.03	2,551 (20.30%)	2.23	6,261 (49.81%)	4.80	12,569
Total	13,382 (32.34%)	—	8,126 (19.64%)	—	19,875 (48.03%)	—	41,383

Percentages in the three platform rows are calculated within platform corpora and are based on the 41,383 comments assigned to the six substantive higher-order thematic groupings. Adjusted standardized residuals indicate deviations from expected counts under independence; positive values indicate over-representation and negative values indicate under-representation relative to the pooled distribution. Pearson χ²(4, *N* = 41,383) = 343.43, *p* < .001; Cramér’s *V* = .064. Percentages may not sum to 100% because of rounding. Values shown in the table are based on the primary consensus coding; alternative coding specifications are reported in S1 File.

Fig 3 visualizes the within-platform distribution of punitive, knowledge-based, and ethical accountability. The thematic and comparative results describe a public judgment structure in which all six thematic groupings and all three accountability functions coexist across communicative settings. Event-node differences concern shifts in the relative salience of thematic concerns, while platform differences concern comparatively small but patterned changes in the composition of accountability functions. The results are therefore consistent with a distributed-accountability interpretation in which punitive, knowledge-based, and ethical demands coexist within each platform environment. The functions do not map one-to-one onto particular platforms.

**Fig 3:**
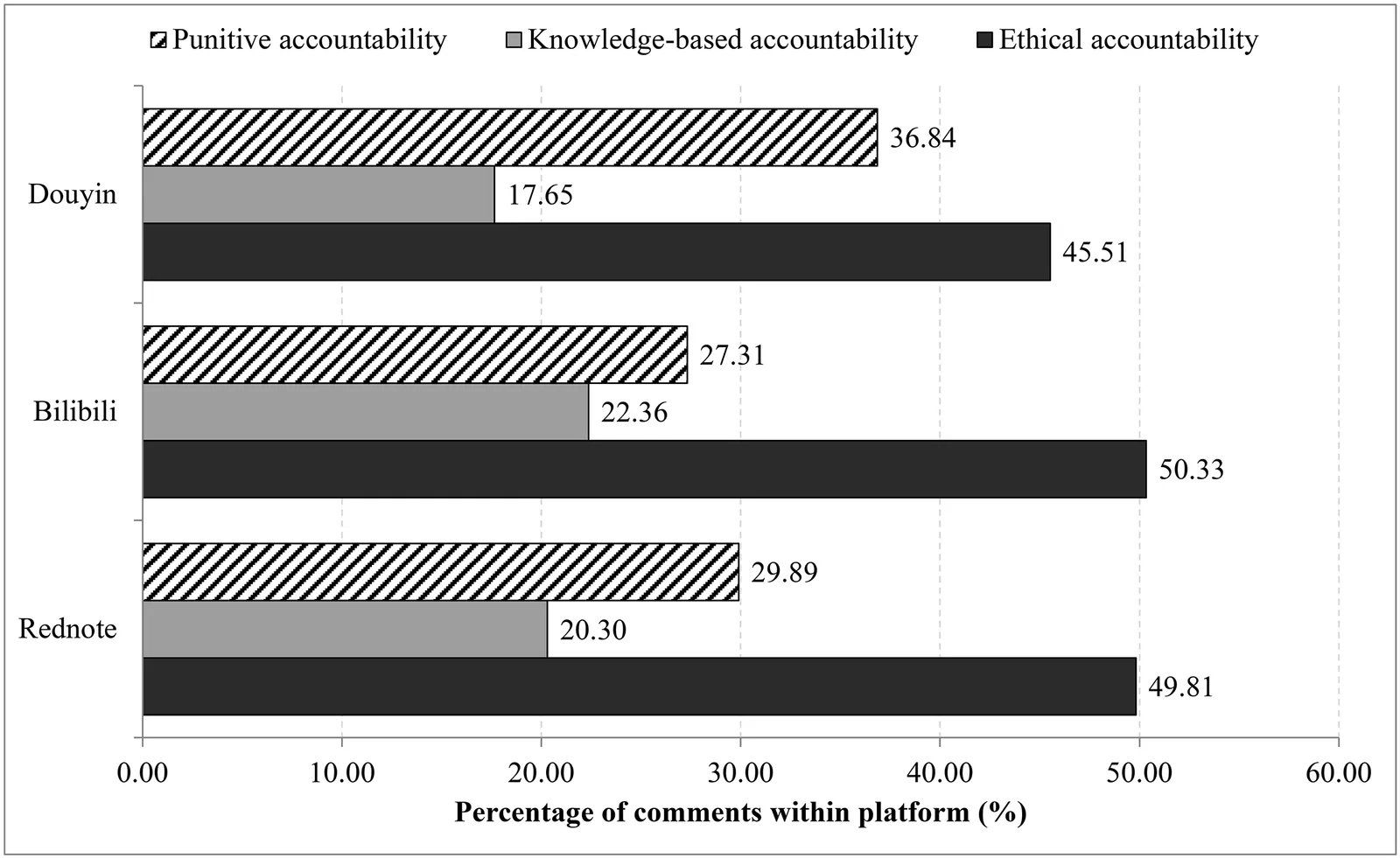
Distribution of accountability functions across platforms. The figure shows within-platform percentages of punitive, knowledge-based, and ethical accountability among the 41,383 comments assigned to the six substantive thematic groupings. All three functions are present in each platform corpus, while their relative composition varies across platforms.

## Discussion

### Affective publicness and crisis visibility

The first layer concerns affective publicness. Topic-representation terms in several groupings foreground ridicule, mockery, absurdity, and spectatorship, and these terms appear alongside terms concerning factual uncertainty and institutional evaluation. No sentiment or emotion measurement was performed. The following reading therefore describes the lexical composition of the topic solution and does not report the prevalence of affect in the corpus. This pattern aligns with research showing that platform publicness is shaped by algorithmic distribution, interface design, and comment interaction [1,49]. Negative emotions can spread rapidly across social boundaries and intensify the circulation of controversy [3].

Affect-oriented topic terms appear within the thematic configurations of all three event-anchored discourse settings. The thematic surroundings of those terms differ across settings. Node A combined moral evaluation of donation obligations with questions concerning the status and authenticity of the donated works. Nodes B and C contained greater relative emphasis on investigation, responsibility attribution, and personalized blame. Legitimacy-oriented topic content was also present across all three event-anchored settings. These differences indicate variation in the composition of public judgment across event-anchored discourse settings. They do not describe a linear trajectory of affective change [63,64].

For museums, affective reactions are closely tied to ethical judgment because these institutions carry strong expectations of public entrustment, symbolic value, and professional authority. Topic content concerning donor relationships, restitution, and perceived breaches of stewardship can be interpreted as linking moral evaluation with affective publicness. In this context, affective publicness forms one layer through which perceived breaches of institutional ethics become publicly expressed.

The cross-platform results also caution against assigning a distinctive emotional register to any single platform. The stable cross-platform finding concerns relative composition. The absolute ordering of accountability functions changed across coding specifications. Douyin showed a comparatively stronger punitive orientation, and Bilibili showed a comparatively stronger knowledge-based orientation across the specifications examined. These patterns are consistent with the possibility that content formats, comment interfaces, community conventions, and distribution systems condition which forms of evaluative expression become more visible, but they do not establish that platform architecture determines users’ affective responses. Content format and comment interfaces are directly observable characteristics of the three platforms, while community conventions and expressive habits provide plausible interpretations consistent with prior research [4,65]. Algorithmic distribution may also affect visibility, but recommendation processes were not observed directly in the present study. Algorithmic effects are therefore treated as a possible mechanism. They are not an empirically identified cause. Affective publicness is therefore best understood as a cross-platform layer of public judgment that becomes articulated through different combinations of moral evaluation, responsibility attribution, and institutional critique.

### Punitive accountability and responsibility attribution

Punitive accountability helps explain how public dissatisfaction can become a structured pursuit of responsibility. Expressions calling for strict investigation, investigation teams, insider theft, and cross-jurisdictional inquiry appeared frequently across the corpus, with investigation and punitive accountability accounting for a larger share of substantive-topic comments in Nodes B and C than in Node A. At the platform level, punitive accountability was relatively over-represented in the Douyin corpus. Punitive accountability is treated here as the accountability function through which the attribution of responsibility becomes action-oriented. It translates blame into demands for investigation, sanction, and visible correction. As prior research has shown, the intensity of attribution shapes anger, loss of trust, and punitive expectations, while higher perceived responsibility increases demands for stronger organizational accountability [12,38].

Responsibility-oriented topic content appeared within each event-anchored discourse setting, although the measured thematic composition differed across nodes. Node A combined responsibility claims with comparatively greater attention to donation ethics and the status of the donated works. Node B showed a stronger concentration of investigation-related discourse, while Node C contained the highest relative share of investigation and punitive accountability and also comparatively elevated personalized blame. These contrasts indicate that responsibility attribution coexisted with different issue emphases across the three nodes [14,42]. They do not constitute a directly observed temporal progression.

The platform comparison likewise revealed relative differences without a one-to-one correspondence between platform and accountability function. Punitive accountability accounted for 36.84% of classified Douyin comments and was strongly over-represented relative to the pooled distribution. Its corresponding shares were lower on Bilibili (27.31%) and Rednote (29.89%), where punitive accountability was under-represented relative to expectation. Bilibili instead showed a relative concentration of knowledge-based accountability. Across the three corpora, responsibility claims kept questions of institutional answerability visible [9,41]. Punitive accountability mattered because it transformed diffuse dissatisfaction into more explicit demands for institutional answerability and corrective action. Expressions invoking insider theft, unresolved closure, or the necessity of strict investigation connected the discrete operational problem to broader judgments of managerial disorder and institutional failure [66]. Responsibility claims extended from specific individuals to museum management and broader cultural-heritage governance structures, consistent with research showing that high-intensity attribution can spill over into wider judgments about related organizations and institutional arrangements [67]. Interpreted through the responsibility-attribution lens, the topic structure suggests that public accountability can extend from particular actors to institutional procedures and governance capacity, while the measured visibility of punitive demands varies across communicative settings.

### Legitimacy pressure and institutional questioning

Responsibility pursuit in platform comments did not stop at identifying specific actors. It extended into broader questioning of institutional procedure, credibility, and governance capacity. Under the primary consensus coding, trust erosion and legitimacy accounted for a substantial share of the thematic distribution across all three event-anchored discourse settings and was the largest grouping overall. The sensitivity analysis shows that its absolute prevalence depends strongly on the classification of Topic 0. The more defensible interpretation therefore concerns the presence of legitimacy-oriented topic content across the corpus, not the rank ordering of G4 relative to the other thematic groupings.

For cultural institutions, legitimacy concerns more than image. It depends on the capacity to sustain professional authority, public support, and social justification [5,8]. Once a crisis affects donation relationships, collection management, and internal governance, a case-specific controversy can escalate into broader judgments about institutional standing [7,29]. Cultural institutions are especially vulnerable because their standing depends heavily on the balance among professional authority, public mission, and institutional norms [15,30]. Topic content concerning trust, institutional credibility, perceived cover-up, and delayed justice provides an empirical basis for interpreting legitimacy questioning in the corpus, including evaluations of whether the museum remained a credible custodian of donated cultural property. Expressions of trust collapse and reluctance to donate extend the object of evaluation beyond short-term reputation to questions of custodial standing, institutional credibility, and the durability of public trust. Transparency, accountability, and sustained responsiveness are therefore central to legitimacy in contemporary cultural institutions [7].

Platform comments functioned as a mediating space between the attribution of responsibility and legitimacy pressure, carrying criticism beyond the immediate event and into broader evaluations of governance capacity, public standing, and institutional fitness. Legitimacy-oriented topic content appeared across all three event-anchored settings, indicating that institutional credibility was not confined to discussion surrounding the final official findings. This content appeared alongside themes concerning donation ethics, authenticity disputes, investigation demands, and personalized blame, providing a basis for interpreting legitimacy questioning as one dimension of the broader topic structure. For cultural policymakers and governance boards, this pattern suggests that legitimacy repair may require public explanation and investigation together with visible attention to collection governance, donor relations, and institutional transparency. Comparable museum crises suggest that legitimacy repair may require public explanation, independent review, and visible governance reform [68,69]. These examples are illustrative only and do not imply that comparable institutional responses would produce the same effects in the present case. The analysis does not establish whether particular institutional responses caused the observed legitimacy concerns, nor whether those concerns increased or declined over time. It shows only that legitimacy-oriented topic content was present across the three event-anchored source-content groups; the analysis does not establish its prevalence as a separately measured dimension.

### Distributed accountability across platform environments

The cross-platform comparison shows that the same controversy did not produce a homogeneous accountability profile. All three accountability functions appeared in every platform corpus, while their relative weighting differed across platforms. The absolute ranking of the three functions was sensitive to coding specification, particularly to the classification of Topic 0. The more stable finding was the cross-platform compositional pattern. Douyin consistently showed a relative punitive concentration. Bilibili consistently showed a relative knowledge-based concentration. The platform–function association remained statistically significant across the alternative specifications, with Cramér’s *V* ranging from  .052 to  .070. This concurrent presence and uneven distribution of accountability functions is what the article conceptualizes as distributed accountability.

The absence of a one-to-one correspondence between platforms and accountability functions is itself an important finding. No platform corpus was organized around a single accountability function, and the relative weighting of the three functions varied across the platform corpora. Douyin’s stronger punitive orientation and Bilibili’s stronger knowledge-based orientation represent comparative differences in relative composition. Neither orientation implies an exclusive correspondence between a platform and an accountability function. Rednote, meanwhile, showed more moderate departures from the pooled distribution than Douyin. Treating accountability function and platform environment as separate analytical dimensions makes mixed profiles observable. The analysis does not impose a predefined functional category on any platform.

This finding deepens existing understandings of platform public discourse. Platform publics are continuously organized through comment culture, content form, and community norms [4,49]. Platform environments provide different communicative conditions under which particular formulations of accountability may become more visible, repeatable, or salient [1,70]. Existing research provides several platform-specific mechanisms that can contextualize these differences. On Douyin, visibility is organized around engagement-sensitive recommendation and short-form production conventions that encourage creators to optimize immediate attention, retention, and algorithmic legibility [71]. This visibility environment offers one plausible context for the relative punitive concentration observed in the Douyin corpus. On Bilibili, video-centered participation has developed alongside distinctive community conventions, including time-synchronized danmu practices and ritualized forms of collective commentary that support interpretive and participatory community formation [53]. The present dataset contains top-level comments; danmu comments were not analyzed. Danmu practices form part of the wider communicative environment in which Bilibili users encounter and discuss public issues. Rednote combines user-generated content production with strong search and discovery practices and an explicit cultural emphasis on useful and authentic sharing, creating a communicative environment in which experiential accounts, practical information, and credibility judgments can become salient [72]. These mechanisms are literature-grounded interpretations of the platform environments and were not measured directly in the present study. The small platform–function effect sizes therefore support a contextual interpretation in which the observed distributions are compatible with differences in platform visibility and interaction conventions but do not identify the causal contribution of recommendation systems, community norms, or user composition. Platform moderation also conditions what can be observed. The corpus contains only comments that were publicly visible and retrievable at the time of collection. Comments removed, collapsed, or otherwise made unavailable before collection are not represented in the dataset. Cross-platform differences therefore describe retrievable public discourse and may reflect moderation and visibility practices as well as differences in user expression.

The findings consequently shift the analytical emphasis from platform-specific accountability to the organization of multiple accountability functions within and across platform environments. These forms of accountability operated in parallel across moral, procedural, and professional dimensions, while their relative weighting varied across platform-specific corpora. Distributed accountability, therefore, captures the concurrent presence and uneven distribution of distinct accountability functions across platform environments. Its value lies in showing that a platformized institutional crisis cannot be addressed through a single response logic, since punitive, knowledge-based, and ethical accountability demand different forms of justification, evidence, sanction, and relational repair.

### Function-based and platform-mediated accountability

The findings should be interpreted through a function-based and platform-mediated lens. Punitive, knowledge-based, and ethical accountability are analytically independent accountability functions. Content format, event context, community conventions, and platform distribution systems may condition their relative visibility and salience. The observed platform–function association was small, which reinforces the need to distinguish patterned variation in relative salience from platform determination. This distinction matters because a platform-determined reading would lock the framework into the specific Chinese platform ecology examined here. A function-based and platform-mediated reading allows the framework to be tested across other platform environments and institutional contexts.

Each function operates independently of any particular platform as an analytical category. Each operates with its own evaluative criteria, affective register, and demand structure. Punitive accountability concerns sanctions and the enforcement of consequences. Knowledge-based accountability concerns procedural propriety and the integrity of institutional reasoning. Ethical accountability concerns moral commitments and the integrity of relational obligations. These distinctions have antecedents in pre-digital accountability scholarship [31,34]. What platformization adds is the concurrent production of these differentiated forms at scale across multiple communicative environments. Platform environments remain analytically relevant because the relative salience of these functions differs across platform-specific corpora. The present findings demonstrate patterned association without deterministic correspondence. The cross-platform differences concerned relative weighting within a shared three-function structure. The results do not support a one-to-one correspondence between platforms and accountability functions. Platform environments may therefore mediate or amplify the relative visibility of particular accountability demands without determining which functions are present or dominant. Such mediation may operate through content formats, distribution systems, interface structures, community conventions, and the composition of visible participation, although the present design does not identify the independent contribution of any one mechanism. Accordingly, the framework separates the substantive function of an accountability demand from the communicative environment in which that demand becomes visible. It therefore preserves the analytical independence of accountability functions while allowing platform environments to be examined as conditions associated with their relative distribution. This separation makes the framework empirically testable across different platform ecologies and institutional settings.

The broader relevance of the framework can be considered through institutional controversies in other national and organizational contexts. The 2023 disclosure of long-term theft from the British Museum provides a useful comparative reference because it likewise concerned custodial responsibility, collection management, and institutional trust. The controversy generated questions about leadership accountability, inventory and security procedures, and the museum’s wider claims to custodial authority. These dimensions correspond conceptually to the punitive, knowledge-based, and ethical demands distinguished in the present framework, with calls for consequences corresponding to punitive accountability, scrutiny of inventory, documentation, and security procedures to knowledge-based accountability, and debates over custodial legitimacy and restitution to ethical accountability. The comparison does not imply that the distribution of these functions across English-language platforms reproduces the pattern observed in the Nanjing Museum case. The comparison illustrates how the three-function framework may travel to another museum crisis in which institutional legitimacy rests heavily on custodial competence and public trust [68,73].

The Sackler donation controversy offers a complementary extension because the locus of accountability differs. The present case centers on the responsibilities of a museum toward donated cultural property and donor relations. The Sackler controversy concerned the ethical implications of museums accepting and publicly recognizing donations associated with contested sources of wealth [74]. Demands to remove donor names can be understood as punitive in the sense that they seek visible institutional consequences; scrutiny of due-diligence and donation-acceptance procedures raises knowledge-based questions about institutional decision making; and disputes over whether such funding is compatible with museums’ public missions raise ethical questions about legitimacy and moral obligation. The comparison suggests that distributed accountability may be useful for custodial controversies and for donor-related crises in which different forms of institutional responsibility become simultaneously salient.

The framework may also extend beyond cultural institutions. Universities, healthcare institutions, public-service organizations, and other trust-dependent organizations may similarly confront functionally differentiated accountability pressures that require different forms of institutional response. This possibility indicates the comparative scope of the framework but does not establish its generalizability. The empirical contribution of the present study remains the Nanjing Museum case, while the broader claim is that distinguishing punitive, knowledge-based, and ethical accountability provides a portable analytical vocabulary for examining how different forms of public demand coexist in institutional crises across contexts.

The position carries several implications. Theoretically, it locates distributed accountability as a property of platformized public judgment, not of any specific platform ecology. The concept, therefore, offers a basis for comparative analysis across national contexts and institutional types whose legitimacy depends on sustained public trust. Methodologically, productive cross-context comparison should focus on identifying the conditions associated with the relative salience of particular accountability functions. Comparative studies across platforms such as TikTok, YouTube, and X could examine whether the coexistence of accountability functions observed here persists under different platform conditions and whether similarly modest or stronger platform-related differentiation emerges. For institutional practice, crisis response strategies should be both function-aware and platform-aware. Platform awareness should not be reduced to assigning a predetermined response type to each platform. An institution facing simultaneous punitive, knowledge-based, and ethical accountability pressures requires distinct response capacities for each, since each demands different forms of justification, evidentiary support, and communicative registers.

### Practical implications for cultural institution governance

The function-based and platform-mediated framework implies that cultural institutions need response capacities differentiated by accountability function, not by platform alone. The present findings reinforce this point because all three accountability functions were present in every platform corpus while their relative weighting differed across platforms. Institutional response should therefore begin by identifying the type of accountability demand being expressed. Response needs should not be inferred from platform identity alone.

To meet punitive accountability pressure, institutions need established protocols for cooperation with regulators and inspection authorities, rapid disclosure of the status of investigations, and visible procedural steps early in a crisis response. Generic regret statements without procedural specificity may be insufficient when public demands center on investigation, responsibility, and visible corrective action. Knowledge-based accountability requires different capacities. Institutions need designated subject-matter spokespersons, publicly accessible provenance and collection management documentation, and transparent explanations of relevant procedures, including deaccessioning, transfer, appraisal, and disposal standards. Ethical accountability shifts the focus toward public mission and donor relationships. Museums need donor relationship protocols that specify notification requirements before disposal, transfer, or deaccessioning actions involving donated objects, along with structured pathways for apology, remediation, and engagement with donor families, descendant communities, or source communities. Ethical accountability accounted for a substantial share of classified comments in each platform corpus under the primary specification. Relational repair, custodial credibility, and the restoration of trust should therefore remain part of crisis response alongside factual clarification and procedural accountability.

At the governance level, museums and supervising authorities need institutional arrangements that can detect and respond to functional differentiation in real time. A cross-platform discourse monitoring function, reporting to senior leadership and governance boards, can identify changes in the relative prominence of punitive, knowledge-based, and ethical accountability demands. Such monitoring should compare the composition of accountability demands within and across platforms. Fixed communicative roles should not be assigned to particular platforms. The small platform–function association observed here indicates that platform differences are meaningful but limited, and that all three accountability functions may require attention within each platform environment. Annual transparency reports on collection management decisions can make procedural conduct verifiable before controversy emerges. Periodic external audits of custodial practices, donation processing, and historical record management can sustain governance credibility across leadership transitions. Crisis response playbooks should be organized by accountability function, with separate response capacities for sanction-oriented demands, evidence-oriented critique, and relationship-oriented repair. Platform-specific adaptation can then be applied at the level of format, timing, and presentation. The underlying response should remain aligned with the accountability function being expressed.

## Limitations

This study has several limitations. The single-case design limits generalizability across different types of cultural institutions and national contexts. The analysis focuses mainly on textual comments and does not incorporate multimodal content or examine account networks, diffusion chains, cross-platform user overlap, or the role of influential users. The analysis identifies differences in observed platform-specific comment distributions but cannot isolate the mechanisms producing those differences. In particular, the study does not have direct access to algorithmic recommendation data and cannot independently estimate the effects of recommendation systems, user composition, community conventions, or other platform mechanisms.

The corpus also contains only comments that were publicly visible and retrievable at the time of collection. Comments removed, hidden, collapsed, or otherwise made unavailable before collection are not represented, and differences in platform moderation and content-visibility practices may therefore affect the observable discourse. Computational topic modeling also remains limited in its treatment of irony, parody, metaphor, and locally specific cultural references, and the analytical results remain sensitive to preprocessing decisions and embedding-model choice. The parameter-sensitivity analysis evaluates the UMAP–HDBSCAN clustering stage and does not directly test the stability of the final reduced 49-topic solution. Although higher-order topic coding used two independent coders and adjudication, the consolidation of first-order topics into thematic groupings remains a researcher-interpreted step. The classification of Topic 0 is a substantive source of uncertainty. Topic 0 was the largest final topic, was disputed by the two coders, and contained semantically heterogeneous material after topic reduction. Sensitivity analyses show that its treatment affects the absolute ranking of thematic groupings and accountability functions, although the principal cross-node and cross-platform compositional contrasts remain stable. Absolute prevalence rankings should therefore be interpreted cautiously. These procedures reduce but do not eliminate interpretive dependence on modeling and consolidation decisions. The observation window ends shortly after the February 2026 official findings and therefore does not capture longer-term changes in institutional reputation, donor relations, governance reform, or public trust.

Finally, the three event nodes classify comments according to the event phase of the source content to which they were attached. The nodes do not form mutually exclusive comment-posting time periods. Comparisons across nodes should therefore be interpreted as differences among event-anchored discourse settings. They are not a longitudinal estimate of changes in public opinion.

## Conclusion

This study examined the Nanjing Museum donated-artifact controversy using 64,349 cross-platform top-level comments to analyze how public judgment forms in platformized institutional crises. The topic solution supports an interpretive reading in which affective expression, responsibility attribution, and legitimacy questioning appear together within the same thematic groupings. The measured findings concern the distribution of six thematic groupings and three accountability functions. All three accountability functions were present in each platform corpus, while their relative weighting differed across platforms. The absolute ranking of the functions was sensitive to the classification of Topic 0. The relative punitive concentration on Douyin and knowledge-based concentration on Bilibili remained stable across alternative coding specifications. These differences were statistically detectable and limited in magnitude. This pattern supports the interpretation of platforms as mediating conditions. It does not support treating particular platforms as determinants of specific accountability functions. Cultural institutions facing platform crises therefore confront multiple accountability demands with distinct response requirements.

The study makes three contributions. Theoretically, it brings together institutional crisis governance, accountability theory, and platformization theory to conceptualize distributed accountability as the functionally differentiated production of public accountability pressure in platformized institutional crises. Empirically, it shows how six thematic groupings are distributed across event-anchored discourse settings, and how punitive, knowledge-based, and ethical accountability are distributed unevenly across the Douyin, Bilibili, and Rednote corpora without forming one-to-one platform-function relationships. The principal empirical contribution therefore concerns the organization of accountability discourse across platform environments, not a universal ordering of the three accountability functions. Practically, it identifies institutional design measures for museums, supervisory authorities, and comparable trust-dependent organizations, including function-specific response capacities, donor relationship management protocols, cross-platform discourse monitoring, and governance review cycles attuned to punitive, knowledge-based, and ethical accountability pressures. Extending the framework requires cases in other platform ecologies and institutional fields, where the same three functions can be coded and their relative distributions compared.

Platformized crises increasingly place cultural institutions under multiple forms of public judgment at once. Distributed accountability captures this condition and offers a framework for analyzing how differentiated accountability pressures become connected to institutional legitimacy. For cultural institutions, the governance challenge extends beyond responding across multiple platforms. Demands for sanction, evidentiary explanation, and relational repair may coexist and require different forms of institutional response.

## Supporting information

S1 File Supporting data workbook for corpus composition, topic coding, comparative analyses, and sensitivity diagnostics. This Excel workbook provides deidentified aggregate and topic-level data for verifying corpus composition and collection windows, the final 49-topic solution, original Chinese and English topic-representation terms, independent validation-round coder assignments and adjudicated thematic coding, the higher-order coding framework, theme- and function-level intercoder reliability, event-node and platform comparisons, Topic 0 reduction-lineage diagnostics, coding-sensitivity analyses, outlier handling, and UMAP–HDBSCAN parameter-sensitivity results. It contains no raw comment text, representative quotations, user or profile identifiers, comment or source-content identifiers, URLs, or precise record-level timestamps.
(XLSX)

S2 File Methods, data availability, and privacy protection protocol. This supporting text documents the corpus-construction rules, embedding preparation and topic-representation procedures, BERTopic model specification and parameter-sensitivity analysis, the two-stage higher-order thematic coding and validation procedure, function-level reliability and coding-sensitivity procedures, the software environment, reproducibility boundaries, data-availability arrangements, privacy protections, and ethics protocol.
(DOCX)

S3 File Analysis code, deidentified analytical data, and reproducibility materials. This ZIP archive provides the author-generated code documenting corpus construction, embedding preparation, BERTopic modeling, outlier reassignment, topic reduction, topic-representation refinement, parameter-sensitivity analysis, higher-order thematic coding, intercoder reliability, coding-sensitivity analysis, RQ1–RQ3 comparative statistics, and source-data generation for Figs 2 and 3. The data/ directory contains three deidentified analytical files, namely comment-level analytical assignments, frozen coder assignments, and public topic-representation data. Scripts 07–12 are executable using the deidentified files included in the package and reproduce the reported downstream reliability, mapping, sensitivity, comparative, and figure-source results. Scripts 01–06 document the restricted text-modeling workflow but require the original third-party user-generated comment corpus and associated restricted inputs for exact execution. The package contains no verbatim user comments, user or profile identifiers, comment or source-content identifiers, URLs, precise record-level timestamps, or cached semantic embeddings.
(ZIP)
