# Supplementary material for: Cross-platform public judgment in a cultural heritage crisis: A topic modeling study of the Nanjing Museum donated-artifact controversy
Source: PLoS One. 2026 Oct 9;21(10):e0360658. doi: 10.1371/journal.pone.0360658 (PMC13652266; doi:10.1371/journal.pone.0360658)
Supplement: Supplementary file 1 [file pone.0360658.s002.docx]

**S2 File. Methods, Data Availability, and Privacy Protection Protocol**

This supporting text documents the corpus-construction rules, topic-modeling specification, manual thematic coding procedure, software environment, and data-governance protocol used in the study. It complements the aggregate and topic-level verification data in S1 File and the analysis code, deidentified analytical data, and reproducibility materials in S3 File.

**1. Study design and corpus construction**

The study used an observational computational text-analysis design to examine publicly visible top-level comments associated with selected source content on Douyin, Bilibili, and Rednote. The corpus was organized by the event phase of the selected source content. Nodes A, B, and C are therefore event-anchored source-content groups rather than mutually exclusive comment-posting time periods. Node membership was inherited from the source item’s original collection package, and the event dates associated with each Node describe the focus of that collection package rather than publication-date eligibility windows for individual source items or comment-posting cutoffs. Comment posting dates can therefore overlap across nodes. Source discovery and source-item selection were conducted manually. Event-specific Chinese-language search terms were entered in Chinese characters on each platform to identify candidate source content, after which candidate items were screened primarily for substantive relevance to the corresponding event node. The representative search combinations reported in the main article are transliterated in Hanyu Pinyin with English glosses for readability; these transliterations were not the literal strings entered into the platform search interfaces. Visible engagement indicators, including like and comment counts, were considered as additional selection criteria among relevant items. Fifty source entries were retained for each platform-by-node combination. The selected source items were subsequently recorded as platform-specific source identifiers or URLs and supplied to the corresponding collection procedures for comment retrieval. The search terms and source-selection procedure are reported in the main article.

| **Stage** | ***N*** | **Change** | **Rule** |
| --- | --- | --- | --- |
| Raw comment observations | 88,552 | — | All retrieved comment observations across the retained source items. |
| After comment-level deduplication | 75,455 | −13,097 | Duplicates removed using platform + comment identifier. |
| After empty-text and post-cutoff exclusions | 74,895 | −560 | 406 empty-text records and 154 records with posting timestamps beyond the applicable platform-specific analytical cutoffs removed. |
| Locked modeling corpus | 64,349 | −10,546 | Nested replies removed; only publicly visible top-level comments retained. |

Platform-specific analytical cutoffs were defined in the Asia/Shanghai time zone. For Douyin and Rednote, comments posted after 23:59:59 on 11 March 2026 were excluded; for Bilibili, comments posted after 23:59:59 on 12 March 2026 were excluded. The locked corpus contained 28,146 Douyin comments, 17,319 Bilibili comments, and 18,884 Rednote comments. By event-anchored source-content group, Node A contained 25,442 comments, Node B 24,784, and Node C 14,123. The corresponding platform-by-node counts and collection windows are reported in S1 File.

**2. Embedding preparation and topic representation**

Corpus construction and semantic embedding preparation were treated as separate stages. The retained raw comment text was encoded using the pretrained Chinese embedding model moka-ai/m3e-base. No additional text-content deduplication, entity normalization, stopword removal, or word segmentation was applied before semantic embedding. The formal BERTopic rerun reused cached M3E-base embeddings generated from these unchanged retained texts and reordered them to the final corpus using platform and comment identifiers. S3 File includes the original embedding-generation procedure for methodological transparency, while the restricted comment-level corpus and cached embedding arrays are not distributed.

| **Component** | **Specification** | **Role** |
| --- | --- | --- |
| Embedding model | moka-ai/m3e-base | Semantic representation of retained raw comment text. |
| Topic-representation tokenization | jieba.lcut; default Jieba dictionary | Tokenization used for lexical topic representation, not for semantic embedding. |
| CountVectorizer during model fitting | token_pattern=None; lowercase=False; ngram_range=(1,1); min_df=10; max_df= .85 | Construction of lexical term counts for topic representation. |
| Representation refinement | c-TF-IDF + lexical MMR; diversity= .30; unigram/bigram candidates; 122-item Chinese stopword list + lexical filters | Improved interpretability and diversification of displayed topic terms. |

**3. BERTopic specification and sensitivity analysis**

| **Stage** | **Parameter** | **Value** |
| --- | --- | --- |
| UMAP | n_neighbors | 25 |
|  | n_components | 5 |
|  | min_dist | 0 |
|  | metric | cosine |
|  | random_state | 42 |
| HDBSCAN | min_cluster_size | 80 |
|  | min_samples | 10 |
|  | metric | euclidean |
|  | cluster_selection_method | eom |
| Outlier reassignment | strategy / threshold | probability-based / 0.10 |
| Topic reduction | target | nr_topics = 50 |

The initial clustering solution contained 149 substantive topics and 23,593 outlier comments (36.66%). Probability-based reassignment assigned 1,370 of these comments to substantive topics, leaving 22,223 final outliers (34.54%). After topic reduction, the final solution contained 49 substantive topics comprising 42,126 comments, together with one outlier cluster.

Targeted sensitivity analysis varied UMAP n_neighbors (15 and 35) and HDBSCAN min_cluster_size (60 and 100) around the baseline specification while holding the embedding model and other clustering parameters constant. Stability was assessed using adjusted Rand index (ARI), adjusted mutual information (AMI), raw HDBSCAN outlier rates, and ARI among documents classified as non-outliers in both the baseline and alternative specifications. This analysis evaluates the UMAP-HDBSCAN clustering stage before final topic reduction and does not directly test the stability of the reduced 49-topic solution. The numerical sensitivity results are reported in S1 File.

**4. Higher-order thematic coding and comparative analysis**

Higher-order thematic coding followed a two-stage procedure. In the initial framework-development stage, two coders independently reviewed the topic-level materials without access to each other’s assignments and subsequently developed the higher-order framework through comparison and consensus discussion. The resulting framework comprised G1–G6, M, and X and was formalized as a written codebook. The codebook was then fixed and applied in a separate independent validation round to the final 49 substantive BERTopic topics. During the validation round, both coders received the same blinded topic-level materials, including topic size, ranked topic-representation terms, and deidentified representative comments, and did not see one another’s assignments or the later adjudicated mapping. The reported intercoder reliability statistics refer to this validation round.

| **Element** | **Procedure** | **Result / analysis denominator** |
| --- | --- | --- |
| Independent validation coding | Each topic assigned to G1-G6, M, or X using the written boundary rules. | 35/49 agreements; 14 disagreements; raw agreement 71.43%; Cohen’s κ = .6602; bootstrap 95% CI [.5017, .8015]. |
| Validation-round adjudication | Disagreements resolved through discussion followed by final consensus review. | 44 topics assigned to G1-G6; 1 topic to M; 4 topics to X. |
| Function-level reliability | The fixed thematic-group-to-function mapping was applied to the frozen validation assignments. | 38/49 agreements; raw agreement 77.55%; Cohen’s κ = .692. Among the 41 topics for which both coders assigned G1–G6, agreement was 33/41 (80.49%; κ = .706). |
| RQ1 | Describe topic-level and higher-order thematic distributions. | 42,126 comments assigned to the 49 substantive topics. |
| RQ2 | Compare six higher-order thematic groupings across event nodes using within-node percentages. | 42,126-comment substantive-topic corpus; M/X retained in the denominator and reported separately. |
| RQ3 | Compare punitive, knowledge-based, and ethical accountability across platform corpora. | 41,383 comments assigned to G1-G6; chi-square, Cramer’s V, and adjusted standardized residuals. |

The finalized coding framework also specified the accountability function associated with each substantive thematic grouping: G1 (donation ethics and restitution) and G4 (trust erosion and legitimacy) were linked to ethical accountability; G2 (authenticity disputes and circulation) and G5 (historical comparison and media circulation) to knowledge-based accountability; and G3 (investigation and punitive accountability) and G6 (personalized blame and family implication) to punitive accountability. M and X were not assigned to accountability functions and were both treated as a single “Not assigned” category at the function level. Coders assigned thematic-group codes during the validation round and did not independently code accountability functions as a separate variable. Function-level agreement was therefore derived from the frozen thematic-group assignments. The complete final mapping, codebook, intercoder statistics, and adjudication summaries are provided in S1 File.

Because the function-level results depend on the topic-to-group assignments, coding sensitivity was evaluated without changing the locked BERTopic solution. Topic 0 was examined separately because it was the largest final substantive topic, was one of the fourteen validation-round disagreements, and showed semantic heterogeneity in the reduction-lineage diagnostic reported in S1 File. Four specifications were compared for the platform-function analysis: the final consensus assignments (S0), the frozen Coder A assignments (S1), the frozen Coder B assignments (S2), and a specification in which Topic 0 was treated as mixed and excluded from function-level analysis (S3). For RQ1 and RQ2, the primary consensus specification was additionally compared with the Topic 0 exclusion specification. These analyses assess the dependence of prevalence rankings and comparative patterns on coding decisions. The primary specification retains the consensus assignments locked before the sensitivity analyses were conducted, thereby avoiding post hoc redefinition of the primary coding in response to the sensitivity results. Absolute prevalence rankings that change across specifications are therefore not treated as stable findings. Full results are reported in S1 File.

**5. Software environment**

The formal BERTopic modeling workflow, including the baseline model, outlier reassignment, topic reduction, and parameter-sensitivity analysis, was executed in a macOS Apple Silicon Python 3.12.13 environment. The verified runtime versions are listed below.

| **Software / package** | **Version** | **Software / package** | **Version** |
| --- | --- | --- | --- |
| Python | 3.12.13 | numpy | 2.4.3 |
| BERTopic | 0.17.4 | scikit-learn | 1.8.0 |
| umap-learn | 0.5.11 | jieba | 0.42.1 |
| hdbscan | 0.8.41 | openpyxl | 3.1.5 |
| sentence-transformers | 5.2.2 | matplotlib | 3.10.8 |
| transformers | 5.3.0 | plotly | 6.6.0 |
| PyTorch | 2.11.0 | tqdm | 4.67.1 |
| pandas | 3.0.1 | scipy | 1.17.1 |
| pyarrow | 23.0.1 | XlsxWriter | 3.2.9 |

The random seed was fixed at 42 for Python random, numpy, torch, and UMAP where applicable. The verified environment included PyTorch 2.11.0; CUDA and ROCm were unavailable, while Apple Metal Performance Shaders support was available. The pretrained moka-ai/m3e-base embedding model was loaded at Hugging Face revision 764b537a0e50e5c7d64db883f2d2e051cbe3c64c. This exact revision is preserved in S3 File as the reproducibility anchor for the embedding procedure.

During preparation of the reproducibility package, the downstream scripts for intercoder reliability, thematic mapping, coding-sensitivity analysis, RQ1–RQ3 comparative statistics, and aggregate figure generation were re-executed using the deidentified analytical files included in S3 File and reproduced the reported results. Scripts 07–12 are executable from the public package using these deidentified inputs. Scripts 01–06 document the corpus-construction, embedding, BERTopic, topic-representation, and coding-material preparation workflow but require the restricted third-party comment corpus and associated restricted analytical inputs for exact execution.

**6. Public supporting materials and data availability**

The public reproducibility materials are organized into three files. S1 File provides deidentified aggregate and topic-level verification data, including corpus composition, final topic information, original Chinese topic terms, thematic coding, event-node comparisons, platform-function distributions, theme- and function-level intercoder reliability, coding-sensitivity analyses, Topic 0 reduction-lineage diagnostics, outlier handling, and model-parameter sensitivity results. S2 File documents the methodological specification, software environment, data-availability procedures, and privacy protections. S3 File provides the author-generated analysis code and deidentified analytical data supporting the study. It includes the documented corpus-construction, embedding, BERTopic, topic-representation, thematic-coding, reliability, coding-sensitivity, comparative-statistical, and figure-generation workflows, together with the software requirements and reproducibility documentation. Scripts 07–12 can be executed using the deidentified analytical files included in the package. The public S3 File package, including the three deidentified analytical files in its data/ directory, is deposited on Zenodo under DOI 10.5281/zenodo.22509326.

The original comment text is not publicly distributed. Although the analyzed comments were publicly visible on Douyin, Bilibili, and Rednote at the time of collection, they constitute third-party user-generated platform content. Verbatim comments may remain traceable to individual platform users through search or platform retrieval even when usernames and direct identifiers are removed, and the authors do not hold redistribution rights that would permit republication of the compiled raw comment corpus. The restriction does not arise from institutional custody of the raw corpus, and no institutional data custodian, data access committee, or ethics committee can authorize redistribution of third-party platform content on the authors’ behalf. Cached semantic embeddings derived directly from the restricted comment text are likewise not publicly distributed. The public supporting materials contain no verbatim comments, usernames, user IDs, profile information, comment or source-content identifiers, URLs, precise record-level timestamps, or cached semantic embeddings.

Other researchers may independently retrieve publicly visible source content from the original platforms using the search terms, event windows, source-selection rules, and collection procedures reported in the article and supporting materials. Because platform content, availability, search rankings, and moderation status may change over time, exact reconstruction of the historical raw corpus cannot be guaranteed.

**7. Privacy protection and ethics**

Collection was limited to publicly visible material available through ordinary registered platform access. No private messages or restricted-access content were collected, and no access controls were circumvented. Direct account and traceable comment-level identifiers were excluded from analytic outputs and public supporting files; results are reported only in aggregate or topic-level form. The principal privacy risk is that verbatim public comments may remain traceable to individual platform users through search or platform retrieval. Representative expressions reported in the manuscript were therefore translated or paraphrased, and verbatim comment text is not included in the public supporting materials.

Ethical approval for this study was granted on 9 January 2026 by the Chair’s Action of the Faculty Ethics Committee, Faculty of Humanities and Social Sciences, City University of Macau (Approval No. FHSS250233). The approved ethics application included a request for waiver of written informed consent for this non-interventional study of publicly visible online comments. No individual informed consent was obtained, and the study involved no direct interaction with platform users. The study examines public judgment and accountability pressure and does not adjudicate the legal or factual responsibility of individual actors.
